# The Development and Testing of an Assessment Scale for Insufficiencies in Family Resilience

**DOI:** 10.3390/nursrep15050145

**Published:** 2025-04-27

**Authors:** Naohiro Hohashi, Natsumi Kijima

**Affiliations:** Division of Family Health Care Nursing, Graduate School of Health Sciences, Kobe University, Kobe 654-0142, Japan; nkijimaa@gmail.com

**Keywords:** assessment scale for insufficiencies in family resilience (IFR), concentric sphere family environment theory, family nursing, family resilience, scale development

## Abstract

**Background and purpose:** When a family becomes aware of family symptoms, family resilience is defined as its power to autonomously and actively improve its own family functions. A quantitative assessment of family resilience is essential in the practice of family nursing. The purpose of this study was to develop a self-assessment scale for family resilience based on the theoretical foundation of the Concentric Sphere Family Environment Theory (CSFET), and to examine the scale’s reliability and validity. **Methods:** Based on 23 categories obtained from previous studies clarifying family conditions demonstrating family resilience, the Assessment Scale for Insufficiencies in Family Resilience (IFR), consisting of 21 items, was developed through content validity examinations by an expert committee and through face validity examinations for family members. The reliability and validity of the IFR were examined for families with children or with family members requiring care. **Results:** Temporal stability over a 2-week interval, which was assessed in 26 subjects, was supported by a high and significant correlation coefficient. The following statistical analysis was performed based on the responses of 206 subjects. The Cronbach’s alpha coefficient showed high internal consistency reliability. The total IFR score showed a moderately significant correlation with the family function score and the family support demands score, demonstrating acceptable criterion-related validity. Exploratory factor analysis confirmed the fit of a five-factor model based on the five systems of CSFET, and construct validity was supported. **Conclusions:** The reliability and validity of the IFR, which is composed of five factors and 21 items based on the CSFET, were confirmed, making the IFR a viable self-assessment scale for determining the level of family resilience.

## 1. Introduction

Resilience is a concept that has been studied extensively in the field of psychology to explain the process, ability, and outcome of experiencing a maladaptive state in a difficult situation, recovery from that state, and returning to an adaptive state [1]. The concept of resilience is applied not only to individuals but also to families, and an approach based on family resilience enables families to withstand and recover from the detrimental challenges they face. While some families are devastated by crises and persistent stress, others become stronger and more resourceful [2].

Originally, resilience was thought to be an innate characteristic of individuals, and was usually viewed in terms of personality traits and coping strategies that enable children and adults to overcome calamitous life experiences. Research into family resilience was initiated by Walsh [2], whose concept of family resilience refers to the ability of the family as a functional system to withstand and recover from adversity. Walsh states that a fundamental premise of family systems theory is that serious crises and persistent life challenges affect the family as a whole and, as a result, key family processes mediate the adaptation or maladaptation of individual family members, their relationships, and the family unit [3].

Scales for measuring family resilience include the Family Resilience Inventory (FRI) [4] and the Family Resilience Scale (FRS) [5]. Both scales were developed based on Walsh’s concept of family resilience [2]. The subjects of the study, however, were not families experiencing difficulties, but highly educated university students and working adults, making generalizations difficult. Furthermore, the items in both scales are limited to measuring strength within the family.

Another family resilience measurement scale is the Family Resilience Assessment Scale (FRAS) [6]. Based on Walsh’s theory, this scale captures family resilience in a multidimensional manner. However, the large number of items (54) places a heavy burden on respondents, making use more difficult. To this end, a 4-item Family Resilience Scale based on the FRAS was developed [7], but because it has too few items, it may not be capable of fully evaluating the broader aspects of family resilience. In addition, the Family Hardiness Index (FHI) [8], which captures one aspect of family resilience—namely, the family’s resistance and resilience to difficulties—is also in use. It has been pointed out that the FHI mainly focuses on the family’s internal resources and does not adequately reflect external factors such as social support. In evaluating family resilience, therefore, the development of a concise and comprehensive scale that clearly reflects the theoretical framework is desirable.

Although the strength of the family as a whole and the interactions between family members are important, families exist within society and function while interacting closely with those outside the family, so it is essential to consider the family’s relationship with the external environment. When measuring family resilience, therefore, a scale is needed that can also measure the strength the family exerts in relation to the external environment. We believe that through the development of a scale that can evaluate a lack of family resilience, it will be possible to screen families that require family nursing.

The Concentric Sphere Family Environment Theory (CSFET) [9] proposed by Hohashi regards the family in the context of interaction/transaction with the internal and external environment, and is suitable as a theoretical foundation for developing a scale that can measure family resilience from a multifaceted perspective. Hohashi defines family resilience as “when a family becomes aware of family symptoms, its power to autonomously and actively improve its own family functions” [10]. In this study, we decided to use CSFET as a theoretical framework, with the family conditions demonstrating family resilience extracted from our previous study [1] as an item pool to develop a new questionnaire to measure family resilience and examine its reliability and validity. In addition, since the concept of resilience includes the process of recovery, it is necessary to conduct research that takes into account the passage of time [11]. In CSFET, the family chrono environment system enables understanding of the family from the perspective of the temporal axis as well.

An approach to family resilience involves a developmental rather than cross-sectional view of family challenges and responses, and examines how resilience changes through various stages of adaptation and the life cycle [2]. Therefore, we believe that a temporal perspective is necessary when considering family resilience, just as it is necessary for individual resilience.

Based on the above, this study aimed to develop the Assessment Scale for Insufficiencies in Family Resilience (IFR), a scale for self-assessing the level of development of family resilience, using CSFET as a theoretical framework, and examining its validity and reliability.

### 1.1. Theoretical Framework

#### 1.1.1. Five CSFET Systems

Five systems based on CSFET are as follows [10]. These are classified as the subscale of IFR.

- The supra system: The outer frame that creates the family environment system, which is directly or indirectly related to other family environment systems, and encompasses the family environment in its entirety.
- The macro system: The family members’ sphere of daily activities that is distant from the family system unit, based on comprehensive physical/objective and psychological/subjective assessments.
- The micro system: A familiar area in the neighborhood of the family system unit, based on comprehensive physical/objective and psychological/subjective assessments.
- The family internal environment system: The family environment system that exists within the family system unit, which is the area within the family system unit where individual family members interact with each other.
- The family chrono environment system: A concept used to indicate the process of temporal change and the transformation of the family internal environment system, the family external environment system, and the family system unit in a time frame from the past to the future.

#### 1.1.2. Characteristics of CSFET

**Figure 1:**
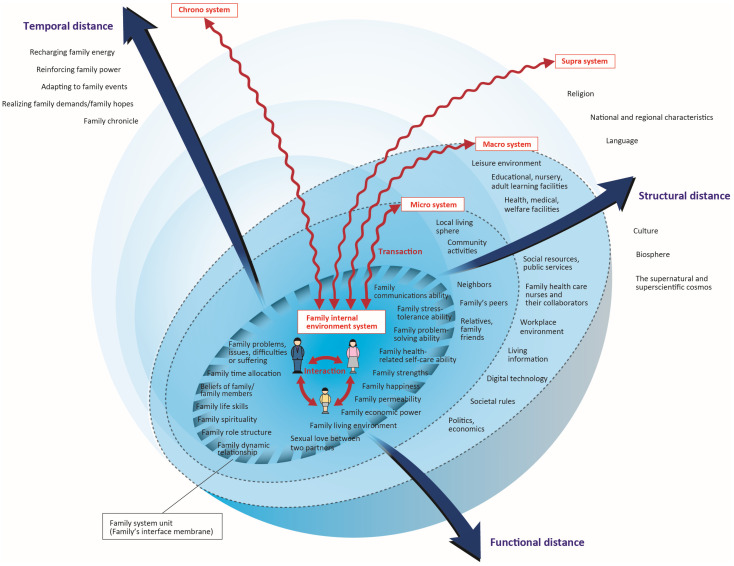
Model diagram of Concentric Sphere Family Environment Theory (CSFET) (Ver. 3.4).

SFET is a middle-range family nursing theory that focuses on the family environment which affects the well-being of the family system unit [9]. In CSFET, the family is considered to be a single unit, but also an interacting system, and the family is treated as a family system unit. The family system unit interacts/transacts with the concentric and spherical family environment that extends inside and outside of it. The family environment is composed of five systems. These systems exist in a three-dimensional logical space formed by three relational axes (structural distance, functional distance, and temporal distance) (Figure 1).

CSFET does not view the family in isolation, but rather considers the family in the context of external family environments such as the nation, society, and region, as well as temporal environments such as the past and future. In particular, family resilience is based on the premises that the family is repairable, that the family grows through the combined efforts of family members when faced with difficulties, and that the family can be strengthened through adversity, despite trying circumstances [12]. In other words, family resilience develops as a family grows and develops through experiences. The CSFET’s family chrono environment system is therefore an important concept for assessing family resilience.

## 2. Methods

We examined content validity and face validity for the item pool based on CSFET when developing the IFR. We then examined test–retest reliability and internal consistency reliability as reliability measures, and criterion-related validity and construct validity as validity measures.

### 2.1. Development of the Scale

#### 2.1.1. Content Validity

As a result of previous research [1], 23 categories were identified as family conditions demonstrating family resilience. Using these as the item pool for the IFR, we repeatedly held expert meetings in which 15 family nursing experts who were members of our laboratory (12 researchers and 3 practitioners) participated to evaluate content validity. With an emphasis on use in clinical settings and to minimize the burden on respondents, we kept the number of items as small as possible and ascertained that the questions were concise. In addition, we considered what each IFR item measured, the relationships between items, whether there were any ambiguous or inappropriate expressions, and so forth, and while confirming that each item measured a single concept, we repeatedly revised and deleted items, improving and revising them, resulting in an IFR with 21 items.

#### 2.1.2. Face Validity

To examine face validity, purposive sampling was used to request the cooperation of 12 families with a member with intellectual disabilities. Families with a member with intellectual disabilities may be able to demonstrate family resilience by confronting various family symptoms (such as family-perceived problems, issues, difficulties, or suffering). After answering the 21-item IFR, a free-form comment section was provided to obtain feedback on the content of the IFR, its readability, and overall impressions. Based on these comments, the items and scale were reconsidered, and the final version of the IFR was developed into a self-administered questionnaire consisting of 21 items. The mean time (SD) for the 12 families to complete the IFR was 5.3 min (2.6), which is regarded as a relatively short time.

#### 2.1.3. Development of the IFR

Family resilience is a subjective concept, and whether it is sufficient for a family depends upon each family’s values and subjective judgment. In other words, it is difficult to set evaluation criteria for family resilience, and collecting measurements from an objective perspective is not possible. In this study, it was decided to measure whether each target family has achieved the family resilience necessary to overcome the problems, issues, difficulties, or suffering that they face, as a norm-referenced test [13], without setting a standard for family resilience per se.

The 21-item IFR is composed of five subscales based on the contents—one supra-system item, four macro-system items, four micro-system items, nine family internal environment system items, and three chrono-system items. In the IFR, the family’s evaluation of each item is evaluated on a five-point Likert scale [14]. The answer options are “Completely disagree = 1”, “Disagree somewhat = 2”, “Neither agree nor disagree = 3”, “Agree somewhat = 4”, and “Strongly agree = 5”. A five-point Likert scale was used to allow respondents to respond neutrally to each item, and to ensure that they were not obliged to select either a satisfied or dissatisfied position. For items related to work, medical treatment, and children, a column was provided to check INAP (inapplicability). If a family member was not working, did not need to go to the hospital, or did not have children, they were asked to check INAP, and the item was excluded from the scoring.

Because responses also included no response or INAP, in some cases not all 21 items were answered, so the item average was used to calculate the total score and the score for each system. In other words, the “family resilience score” consisted of the sum of the answered items divided by the number of answered items. The item average ranged from 1 to 5 points, and the higher the score, the higher the level of family resilience.

**Figure 2:**
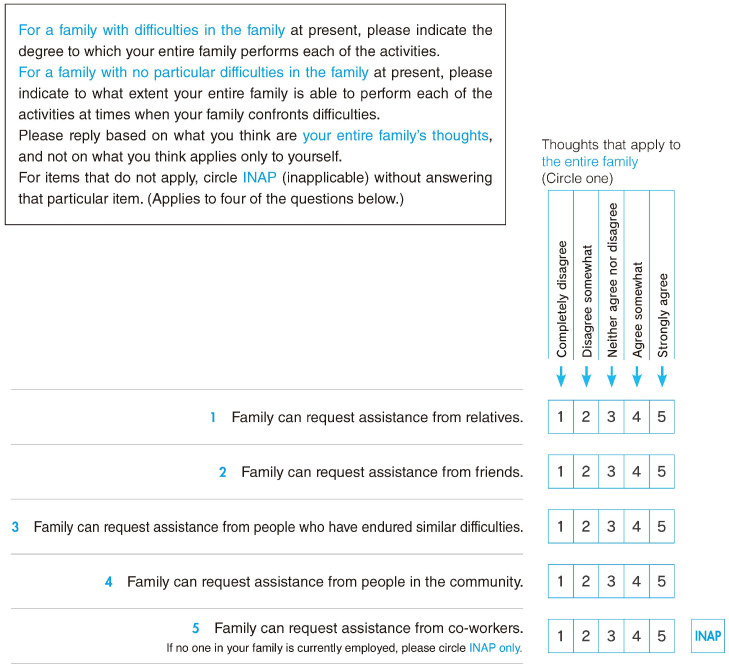
Part of the completed Assessment Scale for Insufficiencies in Family Resilience (IFR).

Through this process, a 21-item IFR was developed based on the five systems of CSFET (Figure 2).

### 2.2. Survey Contents

#### 2.2.1. Basic Attributes, Family Functioning Score, and Family Support Demands Score

The SFE Family Sociodemographics Module (SFE/FSD) was used as the cover sheet [15]. This is a self-administered tool to clarify the minimum basic attributes of a family and the family members, the family functioning status, and the family’s support demands, and is also commercially available.

The basic family attributes were the respondent’s gender and age, family structure, whether or not family members had experienced illnesses, family income, whether or not the respondent was employed, and the respondent’s educational background.

The family functioning status is a result of the family’s subjective perception of the quality of the relationship between the family and family members, and the quality of the relationship between the family and the external environment [10]. Participants were asked to answer using a number ranging from “My family on the whole does not function well at all = 1” to “My family on the whole functions very well = 5”. The higher the family functioning score, the higher the family function.

Family Support Demands is a measure to gauge the extent to which a family needs external support for the problems, issues, difficulties, or suffering they are facing [15]. Respondents were asked to answer using a number ranging from “Family as a whole does not need any support/assistance = 1” to “Family as a whole is in great need of support/assistance = 5”. A higher Family Support Demands score indicates greater family support needs. A completed IFR is available in the Supplementary Materials.

#### 2.2.2. Participants

Families with children and family members who need care were targeted as these are likely to have declining family resilience [1]. Families were recruited in two cities in Japan, one in a rural area and one in an urban area. In the rural area, kindergartens (three facilities), centers for early childhood education and care (two facilities), and daycare facilities (nine facilities) were recruited in City A, a municipality on an isolated island with a population of approximately 33,000. In the urban area, a center for early childhood education and care (one facility) was recruited in City B, an urban municipality with a population of approximately 1.509 million.

### 2.3. Data Analysis Method

Statistical analysis was performed using IBM SPSS Statistics Ver. 28 (IBM Corp., Armonk, New York, NY, USA). Confirmatory factor analysis (CFA) was performed using Amos Ver. 24 (IBM Corp.). As for missing data, when necessary, a series average was imputed using SPSS.

First, the basic attributes of the participating families were calculated. To examine the consistency of the IFR, intraclass correlation coefficients (ICC) were calculated for all items and for each of the five systems. An ICC between 0.6 and 0.8 was interpreted as near agreement, and 0.8 or higher as perfect agreement [16].

#### 2.3.1. Examination of Reliability

To examine the temporal stability of the IFR, a test–retest method with a 2-week interval [13] was performed. Two intraclass correlation coefficients of 0.41 or higher were interpreted as having test–retest reliability.

To examine the internal consistency reliability of the IFR, we calculated Cronbach’s alpha coefficients for each of the five systems and for all items. A Cronbach’s alpha of 0.7 or higher was interpreted as having consistency [17].

To examine the discrimination of the IFR, we calculated item-total correlation. This indicates the degree to which each item correlates with the total score, and Spearman’s rank correlation coefficient must be 0.20 or higher [18].

#### 2.3.2. Examination of Validity

To evaluate the criterion-related validity (concurrent validity) of the IFR, we calculated Spearman’s rank correlation coefficient between the family functioning score and the family support demands score and the total IFR score. For Spearman’s rank correlation coefficient, 0 ≤ |r| < 0.2 was judged to be almost no correlation, 0.2 ≤ |r| < 0.4 was judged to be weak correlation, 0.4 ≤ |r| < 0.7 was judged to be moderate correlation, and 0.7 ≤ |r| < 1 was judged to be strong correlation.

The Kaiser–Meyer–Olkin measure of sampling adequacy (KMO) and Bartlett’s test of sphericity were performed on the 21 items of the IFR. A KMO of 0.6 or higher was judged to indicate that the sampling is adequate [18]. Bartlett’s test of sphericity was used to check whether the results were statistically significant (*p* < 0.05) [18], thereby confirming that the variables were sufficiently correlated and suitable for factor analysis.

To confirm the construct validity of the assumed subscales, exploratory factor analysis was performed and, considering the results, a confirmatory factor analysis by unweighted least squares was used to confirm the goodness of fit of the model. A factor loading of 0.3 was used as the criterion for acceptance [13]. The goodness of fit index (GFI), adjusted goodness of fit index (AGFI), normed fit index (NFI), and root mean square residual (RMR) were used to evaluate the model. The fit indices of GFI, AGFI, and NFI range from 0 to 1, and values of 0.95 or higher were considered to indicate an appropriate fit between the observed model and the theoretical model. For RMR, a value of 0.1 or lower was considered to indicate a good fit [13].

### 2.4. Ethical Considerations

This study was conducted after approval by the institutional review board of the researchers’ university (approval number 454-3). The details of the study were explained to the subjects, who were informed that their cooperation in the study was voluntary, and that the information obtained through the questionnaire was anonymous and would not enable identification of individuals. A research consent check box was provided at the beginning of the questionnaire, with participants checking the consent box indicating agreement to cooperate with the study. To further avoid the impression that participation in the study was coercive, the questionnaires were collected in collection boxes or by mail.

## 3. Results

### 3.1. The Demographic Data of the Target Families

A total of 458 questionnaires were distributed, of which 285 (62.2%) were returned. Of these, 206 were accepted as valid responses. In this study, responses with more than 10% of the total items missing or those with the same numbers selected for all items were treated as invalid responses.

#### 3.1.1. Attributes of Families Using Childcare Facilities

Questionnaires were distributed to 296 families using childcare facilities in cities A and B. From these, 167 copies (58.6%) were returned, with 115 valid responses being analyzed (Table 1). The respondents were 108 women (93.9%) and seven men (6.1%), with an average age (SD) of 37.3 years (5.5). In terms of family structure, 107 (93.9%) were nuclear families, and seven (6.1%) were extended families.

#### 3.1.2. Attributes of Families Using Daycare Facilities

Questionnaires were distributed to 255 families using daycare facilities in City A, and 140 copies (54.9%) were returned, with 91 valid responses being analyzed (Table 1). The respondents were 57 women (62.6%) and 34 men (37.4%), with an average age (SD) of 65.9 years (13.8). In terms of family structure, 89 (97.8%) were nuclear families, and two (2.2%) were extended families.

#### 3.1.3. The Attributes of the Families Subject to the Test–Retest

The attributes of the families subject to the test–retest are shown in Table 2. Questionnaires were distributed to 78 families using childcare facilities in City B, and 30 copies (38.5%) were collected, from which 26 valid responses were analyzed. The respondents were 26 women (100%), with an average age (SD) of 38.0 years (4.6). Of these, 26 (100%) were classified as nuclear families.

### 3.2. IFR Score Distribution

The mean (SD) of the total score of the 21 IFR items was 3.60 (1.22), and the mean response for each item ranged from 1.94 to 4.18 (Table 3).

Spearman’s rank correlation coefficient between each item was calculated, and the correlation between items within each system was 0.20 to 0.78 (Table 4).

Spearman’s rank correlation coefficient between each system was calculated. The supra system had a slightly lower correlation with the other systems (the correlation with the macro system was 0.32; the correlation with the micro system was 0.24; the correlation with the family internal environment system was 0.13; and the correlation with the chrono system was 0.04) (Table 5).

#### 3.2.1. Reliability of IFR

The test–retest results showed that the ICC for all items was 0.92, 0.86 for the supra system, 0.90 for the macro system, 0.85 for the micro system, 0.97 for the family internal environment system, and 0.96 for the chrono system (Table 6).

Cronbach’s coefficient alpha was 0.92 for all items, 0.76 for the macro system, 0.84 for the micro system, 0.93 for the family internal environment system, and 0.91 for the chrono system (Table 7).

Item–total correlations ranged from 0.21 to 0.74 (Table 8).

#### 3.2.2. Validity of IFR

Regarding criterion-related validity, a moderately significant correlation was obtained between the IFR total score and the family functioning score, 0.509 (*p* < 0.001), and between the IFR total score and the family support demands score, −0.411 (*p* < 0.001) (Table 9). Note that the correlation with family support demands is negative due to the difference in the direction of the IFR evaluation.

The Kaiser–Meyer–Olkin measure of sampling adequacy was 0.913, and Bartlett’s test of sphericity yielded a chi-square approximation of 2817.838 (degrees of freedom 210, *p* < 0.001), indicating that the data can be analyzed by factor analysis (Table 10).

A principal component analysis with promax rotation was performed, and the number of factors was fixed at four using a scree plot. As a result, the results converged onto four factors—the first factor was the family internal environment system and the family chrono environment system, the second factor was the micro system, the third factor was the macro system, and the fourth factor was the supra system. Because the family chrono environment system exists throughout the other four systems, it is theoretically possible to interpret it as being placed in the same factor as the family internal environment system (Table 11).

The model structure was examined using confirmatory factor analysis with the five factors that are the theoretical structure of CSFET. As a result, the respective values of GFI, AGFI, NFI, and RMR were 0.986, 0.982, 0.981, and 0.061, indicating that the data closely fit the model (Table 12).

## 4. Discussion

### 4.1. Response Results

The required sample size was calculated to be 197 using G*Power Ver. 3 (Universität Düsseldorf), but this study targeted 206 people, which would be an appropriate sample size. The response rate was 62.2%, which exceeds the recommended value of 50% [13], and is therefore regarded as an appropriate sample.

The average score for each item was 3.6, with only “9. Family can obtain support from religion (religious objects, persons or matters)” showing a low score of 1.94. This is thought to be due to the fact that only 26% of people in Japan maintain beliefs in organized religion [19]. However, considering the potential for the use of this item in other countries and regions with a higher percentage of religious believers, it is believed to be advisable that this item not be deleted [20]. In future research in countries and regions beyond Japan, the necessity of including religion as one of the items should probably be considered.

Spearman’s rank correlation coefficient between each item revealed that “9. Family can obtain support from religion (religious objects, persons or matters)” in the supra system had a low correlation with all other items. The particularly low correlation with the family chrono environment system may be due to the fact that religion is universal and has little connection to the concept of a time axis.

### 4.2. Reliability of IFR

In the test–retest method, the number of valid responses was 26, which was slightly low compared to the required sample size of 34, calculated using G*Power Ver. 3. However, the ICC was 0.92 for the 21 items as a whole, and for the five subscales—it was 0.86 for the supra system, 0.90 for the macro system, 0.85 for the micro system, 0.97 for the family internal environment system, and 0.96 for the family chrono environment system—and it was determined that the temporal stability was favorable.

Cronbach’s coefficient alpha for the 21 items as a whole was 0.92, which is above 0.90, indicating that the internal consistency reliability was high [21]. In addition, for the four subscales except for the supra system, which has only one item, the macro system had a high value of 0.76, the micro system had a high value of 0.84, the family internal environment system had a high value of 0.93, and the family chrono environment system had a high value of 0.92, thereby confirming that the IFR had internal consistency reliability.

Regarding discrimination, the item–total correlation of the 21 items ranged from 0.21 to 0.74, with no items below 0.2. It was determined that each item adequately reflected what the scale was measuring.

### 4.3. Validity of IFR

The IFR showed a moderately significant correlation with the family functioning score of 0.51 and with the family support demands score of −0.41. Family resilience is defined as “when a family becomes aware of family symptoms, its power to autonomously and actively improve its own family functions”, and family function is enhanced by family resilience. In other words, family function is not a completely consistent concept with family resilience, but is a related concept. In addition, the definition of family resilience cites “a family becomes aware of family symptoms” as a prerequisite. Although it is not a completely consistent concept with family support demands, which measures the degree to which family nursing is needed, a decrease in family resilience may appear in family support demands, therefore making it a related concept. It was thus determined that the moderate correlation established criterion-related validity.

Exploratory factor analysis revealed a four-factor structure. The first factor was the family internal environment system and the family chrono environment system; the second factor was the micro system; the third factor the macro system; and the fourth factor the supra system—all converging into four factors. The reason why the family internal environment system and the family chrono environment system were included in the first factor is thought to be because they showed a high correlation of 0.82. In addition, the family chrono environment system exists throughout the other systems, and the items in the IFR family chrono environment system—“19. Family can utilize its past experiences”, “20. Family can make time for building good relationships”, and “21. Family can establish goals that are aimed toward solutions”—are items related to the internal family environment, so it was determined that they could be adapted to the CSFET system.

Confirmatory factor analysis was performed using five factors based on the five CSFET systems, and the results were found to be valid, with a GFI of 0.986, an AGFI of 0.982, an NFI of 0.981, and an RMR of 0.061. The factor structure of the IFR was consistent with CSFET, and the construct validity of the IFR was established. From the above, it was determined that the IFR has a five-factor structure based on the five systems of CSFET.

### 4.4. The Characteristics of the IFR and Suggestions for Its Application to Nursing

The 21 items of the IFR cover the five systems of the CSFET—the supra system, macro system, micro system, family internal environment system, and family chrono environment system. It is believed that the IFR can be used to comprehensively measure the resilience of families as they grow and develop while interacting/transacting with various family environments. Unlike existing family resilience scales, the IFR includes not only family resilience within the family, but also family resilience exerted toward the external family environment and the family chrono environment system, and has the characteristic of being able to evaluate the family comprehensively.

Families with a reduced family resilience have difficulty overcoming difficult situations on their own, so family nursing that draws out the strength of the family is necessary. However, it is unclear for what kinds of family condition family nursing should be provided, and it may be difficult for the family themselves to ask for help from a third party. Therefore, families with a lack of family resilience may not be able to obtain support from a third party when they find themselves in a difficult situation, which they may not be able to overcome. The IFR can be completed by the family in a short time, facilitating assessment of the state of family resilience. In addition, because it is a questionnaire, it is more likely that family members will be able to write the truth than if a nurse were to interview them directly. For this reason, the IFR can be said to be an effective family assessment tool, as it allows for a more objective and accurate understanding of family resilience and can lead to early family intervention for families in need of family nursing care.

### 4.5. Research Limitations and Future Prospects

This study was limited to families with child-rearing members and those requiring care, and does not cover all families in which family resilience showed declines. It will be necessary to further evaluate the generalizability of the results to different family attributes, different countries and regions, and environments in which family nursing is actually required. Moreover, although this scale was created with the assumption that it will be used outside of Japan, it will be necessary to examine the reliability and validity of other versions of the IFR apart from the Japanese version.

## 5. Conclusions

This study covers the development of the IFR, a scale for measuring family resilience, based on the theoretical foundation of CSFET. The IFR’s temporal stability, internal consistency reliability, criterion-related validity, and construct validity were established, providing evidence of its reliability and validity. The IFR comprehensively captures the family based on the family environment both inside and outside the family that interacts/transacts with the family, and can be used in research and clinical settings.

## Figures and Tables

**Table 1:** The demographic data of the target families (*n* = 206).

Characteristics	*n* (%)	*M* (*SD*)	Range
Affiliated facilities			
Childcare facilities	115 (55.8)		
Daycare facilities	91 (44.2)		
Sex			
Childcare facilities			
Female	108 (93.9)		
Male	7 (6.1)		
Daycare facilities			
Female	57 (62.6)		
Male	34 (37.4)		
Type of family			
Childcare facilities			
Nuclear family	107 (93.9)		
Extended family	7 (6.1)		
Daycare facilities			
Nuclear family	89 (97.8)		
Extended family	2 (2.2)		
Families with ill members			
Childcare facilities			
Present	6 (5.2)		
Not present	109 (94.8)		
Daycare facilities			
Present	51 (56)		
Not present	40 (44)		
Academic level of respondent			
Childcare facilities			
High school or lower	2 (1.8)		
Occupational school and above	112 (98.2)		
Daycare facilities			
High school or lower	27 (30.3)		
Occupational school and above	62 (69.7)		
Respondent’s employment			
Childcare facilities			
Employed	92 (81.4)		
Not employed	21 (18.6)		
Daycare facilities			
Employed	47 (52.8)		
Not employed	42 (47.2)		
Respondent’s age			
Childcare facilities		37.3 (5.5)	23–66
Daycare facilities		65.9 (13.8)	36–94
Number of family members			
Childcare facilities		4.7 (1.8)	2–13
Daycare facilities		3.4 (2.0)	2–15
Number of children			
Childcare facilities		4.7 (1.8)	1–5
Daycare facilities		0.1 (0.4)	0–3
Family income (in 10,000 Japanese yen)			
Childcare facilities		671.3 (414.6)	216–3096
Daycare facilities		398.8 (321.3)	70–1500

Note: Items not receiving responses were excluded from the analysis. JPY 1 = USD 144 (the exchange rate at the time of the study).

**Table 2:** Demographic data of test–retest subjects (*n* = 26).

Characteristics	*n* (%)	*M* (*SD*)	Range
Sex			
Female	26 (100)		
Male	0 (0)		
Type of family			
Nuclear family	26 (100)		
Extended family	0 (0)		
Families with ill members			
Present	1 (3.8)		
Not present	25 (96.2)		
Academic level of respondent			
High school or lower	4 (16)		
Occupational school and above	21 (84)		
Respondent’s employment			
Employed	19 (76)		
Not employed	6 (24)		
Respondent’s age		38 (4.6)	31–48
Number of family members		4.8 (2.1)	2–13
Number of children		2.3 (0.95)	1–5
Family income (in 10,000 Japanese yen)		684 (243)	300–1210

Note: Items not receiving responses were excluded from the analysis. JPY 1 = USD 144 (the exchange rate at the time of the study).

**Table 3:** Means and standard deviations of IFR scores (*n* = 206).

Item No.	Item (System Name Indicated in Brackets)	*M* (*SD*)	Range
1	Family can request assistance from relatives. [Mic]	3.78 (1.23)	1–5
2	Family can request assistance from friends. [Mic]	2.96 (1.32)	1–5
3	Family can request assistance from people who have endured similar difficulties. [Mic]	2.76 (1.30)	1–5
4	Family can request assistance from people in the community. [Mac]	2.72 (1.23)	1–5
5	Family can request assistance from co-workers. [Mac]	2.87 (1.16)	1–5
6	Family can request assistance from specialists (physicians, nurses, psychological counselors, etc.). [Mac]	3.55 (1.10)	1–5
7	Family can request assistance from kindergartens, nurseries, schools, etc. [Mac]	3.55 (1.23)	1–5
8	Family can seek out and utilize public assistance (e.g., unemployment insurance, nursing insurance, assistance to single parents, facilities for the handicapped, etc.). [Mac]	3.66 (1.17)	1–5
9	Family can obtain support from religion (religious objects, persons or matters). [Sup]	1.94 (1.16)	1–5
10	Family can consult with one another. [Int]	4.17 (1.08)	1–5
11	Family can search out and utilize knowledge necessary for solutions. [Int]	4.02 (1.04)	1–5
12	Family can accept family problems. [Int]	4.18 (0.95)	1–5
13	Family can accept family members with illness or handicaps. [Int]	4.15 (0.84)	1–5
14	Family can supplement roles that are lacking in the family (e.g., child rearing, housework, nursing and/or medical care and parenting). [Int]	3.78 (0.99)	1–5
15	Family can provide assistance to family members having problems. [Int]	3.98 (0.94)	1–5
16	Family can maintain appropriate distance (psychological and physical distance) between its members. [Int]	3.79 (0.90)	1–5
17	Family can obtain beliefs that provide family with spiritual support. [Int]	3.64 (0.97)	1–5
18	Family can work together in mutual cooperation. [Int]	4.00 (0.99)	1–5
19	Family can utilize its past experiences. [Chr]	3.89 (0.99)	1–5
20	Family can make the time for building good relationships. [Chr]	3.77 (1.10)	1–5
21	Family can establish goals that are aimed toward solutions. [Chr]	3.81 (1.22)	1–5
	Total score (item average)	3.60 (1.22)	

Note: Items number 5, 6, 7, and 13 contain a box where inapplicable (INAP) may be selected. Sup = supra system; Mac = macro system; Mic = micro system; Int = family internal environment; Chr = family chrono environment system.

**Table 4:** Correlation matrix between each item of the IFR (*n* = 206).

Item No.	1	2	3	4	5	6	7	8	9	10	11	12	13	14	15	16	17	18	19	20	21
1 [Mic]	1																				
2 [Mic]	0.53 **	1																			
3 [Mic]	0.53 **	0.61 **	1																		
4 [Mac]	0.40 **	0.66 **	0.69 **	1																	
5 [Mac]	0.23 **	0.35 **	0.36 **	0.43 **	1																
6 [Mac]	0.25 **	0.21 **	0.31 **	0.23 **	0.52 **	1															
7 [Mac]	0.36 **	0.40 **	0.47 **	0.39 **	0.49 **	0.58 **	1														
8 [Mac]	0.11	0.11	0.13	0.08	0.26 **	0.55 **	0.34 **	1													
9 [Sup]	0.04	0.17 *	0.16 *	0.25 **	0.24 **	0.25 **	0.20 **	0.22 **	1												
10 [Int]	0.36 **	0.30 **	0.31 **	0.30 **	0.23 **	0.27 **	0.40 **	0.20 **	0.11	1											
11 [Int]	0.31 **	0.28 **	0.32 **	0.37 **	0.29 **	0.27 **	0.40 **	0.32 **	0.13	0.69 **	1										
12 [Int]	0.26 **	0.25 **	0.26 **	0.25 **	0.16 *	0.17 *	0.34 **	0.20 **	0.12	0.61 **	0.60 **	1									
13 [Int]	0.15 *	0.20 **	0.19 **	0.25 **	0.19 **	0.27 **	0.31 **	0.22 **	0.15 *	0.51 **	0.49 **	0.69 **	1								
14 [Int]	0.29 **	0.27 **	0.18 **	0.21 **	0.21 **	0.19 **	0.28 **	0.12	0.06	0.48 **	0.46 **	0.55 **	0.62 **	1							
15 [Int]	0.33 **	0.34 **	0.31 **	0.24 **	0.16 *	0.22 **	0.32 **	0.18*	0.07	0.46 **	0.52 **	0.59 **	0.59 **	0.63 **	1						
16 [Int]	0.31 **	0.31 **	0.27**	0.24 **	0.23 **	0.23 **	0.36 **	0.13	0.02	0.53 **	0.57 **	0.59 **	0.58 **	0.55 **	0.63 **	1					
17 [Int]	0.26 **	0.29 **	0.29 **	0.22 **	0.14 *	0.11	0.32 **	0.16 *	0.14 *	0.49 **	0.53 **	0.60 **	0.53 **	0.39 **	0.50 **	0.61 **	1				
18 [Int]	0.32 **	0.27 **	0.33 **	0.26 **	0.21 **	0.16 *	0.33 **	0.15 *	0.08	0.56 **	0.63 **	0.64 **	0.52 **	0.65 **	0.63 **	0.69 **	0.63 **	1			
19 [Chr]	0.30 **	0.31 **	0.33 **	0.29 **	0.27 **	0.19 **	0.36 **	0.16 *	0.09	0.51 **	0.65 **	0.66 **	0.53 **	0.51 **	0.64 **	0.63 **	0.65 **	0.74 **	1		
20 [Chr]	0.32 **	0.32 **	0.28 **	0.26 **	0.27 **	0.15 *	0.33 **	0.19 **	0.00	0.51 **	0.62 **	0.63 **	0.50 **	0.52 **	0.55 **	0.66 **	0.58 **	0.73 **	0.67 **	1	
21 [Chr]	0.34 **	0.32 **	0.25 **	0.27 **	0.18 *	0.16 *	0.38 **	0.16 *	0.04	0.56 **	0.67 **	0.67 **	0.51 **	0.53 **	0.55 **	0.63 **	0.63 **	0.72 **	0.79 **	0.78 **	1

Note: Sup = supra system; Mac = macro system; Mic = micro system; Int = family internal environment; Chr = family chrono environment system; * *p* < 0.05; ** *p* < 0.01.

**Table 5:** Correlation matrix between each system of the IFR (*n* = 206).

	Sup	Mac	Mic	Int	Chr
Sup	1				
Mac	0.32 **	1			
Mic	0.24 **	0.49 **	1		
Int	0.13 *	0.37 **	0.37 **	1	
Chr	0.04	0.32 **	0.33 **	0.82 **	1

Note: Sup = supra system; Mac = macro system; Mic = micro system; Int = family internal environment; Chr = family chrono environment system; * *p* < 0.05; ** *p* < 0.01.

**Table 6:** Correlation coefficients for IFR scores over a 2-week period (*n* = 26).

System	First Compilation *M* (*SD*)	Second Compilation *M* (*SD*)	ICC
Sup (1 item)	1.81 (1.14)	2.17 (1.18)	0.86 ***
Mac (4 items)	3.50 (1.11)	3.60 (1.12)	0.90 **
Mic (4 items)	3.34 (1.28)	3.67 (1.07)	0.85 ***
Int (9 items)	4.22 (0.81)	4.35 (0.79)	0.97 ***
Chr (3 items)	4.28 (0.77)	4.22 (0.84)	0.96 ***
Total (21 items)	3.81 (1.15)	3.95 (1.08)	0.92 ***

Note: Sup = supra system; Mac = macro system; Mic = micro system; Int = family internal environment; Chr = family chrono environment system; ICC = intraclass correlation coefficients; ** *p* < 0.01; *** *p* < 0.001.

**Table 7:** Cronbach’s coefficient alpha for IFR scores (*n* = 206).

System	Cronbach’s Alpha
Mac (4 items)	0.76
Mic (4 items)	0.84
Int (9 items)	0.93
Chr (3 items)	0.91
Total (21 items)	0.92

Note: Mac = macro system; Mic = micro system; Int = family internal environment; Chr = family chrono environment system.

**Table 8:** Item–total correlations for IFR (*n* = 206).

Item No.	*r*
1	0.48
2	0.55
3	0.54
4	0.55
5	0.44
6	0.42
7	0.57
8	0.30
9	0.21
10	0.69
11	0.74
12	0.68
13	0.64
14	0.60
15	0.66
16	0.70
17	0.65
18	0.73
19	0.74
20	0.70
21	0.74

**Table 9:** Criterion-related validity between family functioning score and family support demands score (*n* = 206).

	*R*
family functioning score	0.51 **
family support demands score	−0.41 **

Note: ** *p* < 0.01.

**Table 10:** Kaiser–Meyer–Olkin measure of sampling adequacy and Bartlett’s test of sphericity (*n* = 206).

Kaiser–Meyer–Olkin measure of sampling adequacy	0.913
Bartlett’s test of sphericity	
chi-square approximation	2817.838
degree of freedom	210
*P*	0.000

**Table 11:** Factor loadings of the exploratory factor analysis (*n* = 206).

Item No.	Factor 1	Factor 2	Factor 3	Factor 4	Communality
18	**0.897**	0.017	−0.075	−0.045	0.78
21	**0.889**	0.035	−0.069	−0.073	0.78
12	**0.861**	−0.040	−0.088	0.160	0.70
19	**0.836**	0.051	−0.019	−0.036	0.73
17	**0.807**	0.055	−0.171	0.139	0.64
20	**0.805**	0.050	0.001	−0.168	0.70
16	**0.802**	−0.009	0.085	−0.184	0.71
13	**0.784**	−0.149	0.097	0.143	0.62
11	**0.759**	−0.011	0.143	0.078	0.67
15	**0.719**	0.067	0.013	−0.043	0.57
10	**0.713**	0.051	0.067	0.073	0.59
14	**0.712**	−0.057	0.080	−0.026	0.51
3	−0.044	**0.854**	0.048	0.035	0.74
2	0.022	**0.853**	−0.052	0.058	0.72
4	−0.017	**0.844**	−0.010	0.172	0.73
1	0.094	**0.699**	−0.062	−0.082	0.52
6	−0.055	−0.024	**0.908**	−0.005	0.78
8	0.081	−0.280	**0.763**	0.189	0.61
5	−0.083	0.285	**0.638**	−0.070	0.57
7	0.103	0.272	**0.590**	−0.096	0.61
9	0.002	0.127	0.056	**0.906**	0.87
Explained variance	9.050	2.605	1.553	0.949	
Explained percentage	43.097	12.407	7.395	4.517	
Cumulative percentage	43.097	55.503	62.898	67.415	

**Table 12:** Results of confirmatory factor analysis (*n* = 206).

GFI	0.986
AGFI	0.982
NFI	0.981
RMR	0.061

Note: GFI = goodness-of-fit index; AGFI = adjusted goodness of fit index; NFI = normed fit index; RMR = root mean square residual.

## Data Availability

The data presented in this study are available on request from the corresponding author.

## Supplementary Materials

The completed IFR can be downloaded for free use at this link: https://e-familynursing.org/assets/IFR_1.4EN.pdf (accessed on 27 March 2025).

## Public Involvement Statement

The families in the community participated on a voluntary basis after being informed of the purpose of the research, and completed a self-administered questionnaire.
